# How Equine-Assisted Activities Affect the Prosocial Behavior of Adolescents

**DOI:** 10.3390/ijerph17082967

**Published:** 2020-04-24

**Authors:** Imre Zoltán Pelyva, Réka Kresák, Etelka Szovák, Ákos Levente Tóth

**Affiliations:** 1 Doctoral School of Health Sciences, Faculty of Health Sciences, University of Pécs, H-7621 Pécs, Hungary; ethyfitwell@gmail.com; 2 Institute of Psychology, Faculty of Humanities and Social Sciences, University of Szeged, H-6722 Szeged, Hungary; kresak.reka@gmail.com; 3 Institute of Sport Sciences and Physical Education, Faculty of Science, University of Pécs, H-7624 Pécs, Hungary; totha@gamma.ttk.pte.hu

**Keywords:** equine-assisted activities, adolescence, behavior problems, prosocial behavior

## Abstract

Multiple studies have investigated the positive effects of human–animal interactions and showed that animal-assisted activities can be successfully used to better human physical and mental health. Equine-assisted activities have also raised considerable attention within the field. Our research focuses on healthy students (aged 14–18) without deviations or special educational needs. We analyze the occurrence of behavior problems and prosocial behavior among adolescents who regularly have interactions with horses, and those who have no connection to horses at all. The subjects of our investigation completed the strengths and difficulties questionnaire (SDQ), and we use a ‘quasi’ 2 × 2 before-after control-impact design to analyze the data. Students studying equine-related vocations and students of other vocations are compared, at the beginning and at the end of their studies. Our results indicate that students of equine-related vocations are more helpful and empathetic, and have fewer behavior problems, than those studying other vocations. There is a negative correlation between prosocial behavior and behavior problems. The development of the prosocial behaviors of students with regular horse–human interactions is more remarkable than of those who have no connection to horses. With these results, we are going to confirm the hypothesis that equine-assisted activities correlate with positive behavioral traits among healthy adolescents.

## 1. Introduction

Adolescence is a time of fundamental physical, mental, and emotional changes, making it really hard for teenagers to cope with life. This is a stage when mental health issues often surface, and if not managed in time, these can lead to delinquency or substance abuse [1], or can cause illnesses later in life [2]. For this reason, exploring ways to protect and help adolescents struggling with psychosocial factors is of high importance, and strengthening prosocial behaviors was found to be one such way [3]. This article describes a nonconventional method that may help youth to cope better in the adult world.

Animal-assisted activities (AAI) have become a popular and legitimate field of research in the last few decades; the number of studies regarding this area has grown, aiming to obtain the scientific evidence that verifies its positive effects on human health [4]. Likewise, the role of equine-assisted activities (EAA) in physical and mental health issues is also getting more attention worldwide. Our study concentrates on the influence of EAA on certain psychological factors. From the end of the 1990s, several investigations were conducted which justified the idea that horses, riding, and equine-assisted activities have a positive effect on a number of behavioral traits, emotional and social skills, and competences. Such favorable effects are the strengthening of positive feelings and optimism [5], improvement of self-confidence, a healthy self-concept, self-acceptance, creativity, and assertiveness, and the decrease of anxiety and isolation [6]. These phenomena help the subtlety of communication, the recognition and conscious control of emotions [7,8], and via these, social integration. Considering personality development, EAA can promote metacognition, self-respect, and developing a responsible lifestyle [9]. In addition to beneficially shaping the sense of self-efficacy, autonomy, and empathy [10], working with horses can also strengthen perceived social support [11], and may also favorably affect cooperation, sociability, and respect [12], in this way promoting the development of personality traits and social skills at the same time.

According to the Professional Association of Therapeutic Horsemanship International (PATH Intl.), “equine-assisted activities include specific activities, …, mounted or ground activities, grooming and stable management, …, in which clients, participants, volunteers, instructors and equines are involved” [13]. During EAA, development and learning happen via controlled horse–human interactions, involving experimental growth [14] based on interspecific relationships [15]. The goal of these activities can be the development of a given skill, such as communication, problem solving or active listening [8,16], the bettering of peer relations, processing and managing emotions in general [17], or even a change of character, in the broad sense of the word [18]. The range of possible subjects is varied and wide, and can include top managers, at risk youth or abused women [19], and even children with a past of gender-based violence [20]. 

Having more than 20 years of experience educating adolescents, students of both equine-related and other vocations, we observed the phenomenon, and now formulate the hypothesis that youths who have a strong relationship with horses and a commitment to horse-related tasks, have fewer behavior problems and stronger prosocial skills (prosocial skills can refer to a wide range of behavioral patterns intended to benefit the other more than the actor [21]; the tool of our investigation—the SDQ questionnaire, to be discussed later—focuses on sharing with and helping others, considering others’ feelings, and being kind to them) than their peers without such engagements. 

The number of studies supporting similar observations and experiences with exact research data—some quoted above—are quite low. Statistically justifying that adolescents who have regular interactions with horses show a significantly more positive behavior than those who have none, and also confirming that EAA is a decisive factor in such behaviors, would make an important contribution to the study, as well as to the understanding of human–animal interactions and their positive effects. Furthermore, this finding would support the need of making horse–human interactions available to a larger number of youths on a more regular basis.

The purpose of this study is to investigate the above described hypothesis; that is, whether a difference exists in the behavior (occurrence of behavior problems and prosocial behavior) between youths studying equine vocations and thereby having regular interactions with horses, as opposed to those who have no relationship to horses at all.

In our research, EAA is used within an educational environment, investigating the effect of time spent with horses on healthy 14–18-year old students of equine vocations. It will be demonstrated that through this school-based intervention—which may be an efficient way of cooperation with other educational and prevention services for the good of adolescents [22]—the prosocial behaviors of the target group will strengthen, which—as described in our opening paragraph—may help adolescents and protect them from psychosocial stressors they have to face in their everyday lives.

## 2. Materials and Methods 

### 2.1. Study Population

Our investigation was based on a cross-sectional study with a control group. Our target group included 14–18-year-old students from 10 agricultural secondary schools in Hungary. They all took part in a four-year horse groom training program. These students had no diagnosed physical or psychological difficulties. Within the curriculum of the schools, they spent two days, that is 9–13 hours, with horses per week. On these occasions, they fed and groomed the horses, cleaned the stable, and worked with the horses on the lunge, from the saddle, and also practiced carriage driving. Later on, these students are referred to as equine students (ES). Members of the control group consisted of students from the same schools who studied non-horse related, agricultural, or food industry vocations (e.g., gardening, animal husbandry, meat processing, baking) and thus, did not take part in any activities involving horses. They make up the other students (OS) group of the study.

### 2.2. Methods

We used the ‘Strengths and Difficulties Questionnaire’ to measure the participants’ social and emotional competences. It was originally developed for 2–17-year-old children, to identify and explore emotional and behavior problems and psychic disturbances, and has been used before with EAA-related studies [11,12,23]. It embraces a wide spectrum of emotional and social competences and is internationally used, being available in many languages, including Hungarian [24]. In the present study, the one-sided self-rated questionnaire for 11–17-year-olds was used. All versions of the SDQ make statements about 25 attributes, some positive and others negative. These 25 items are divided between five scales: (1) Emotional difficulties (ED), (2) behavioral difficulties (BD), (3) hyperactivity (H), (4) peer-relations (PR), and (5) prosocial behavior (PB). Scales (1) to (4) are added together to generate a total difficulty score (TS) (based on 20 items). Subjects have to decide whether each statement is ‘Not True’, ‘Somewhat True’, or ‘Certainly True’ for them. The answers get a score of ‘0’, ‘1’, or ‘2’; at certain statements ‘Not True’ gets the ‘0’ score, at others ‘Certainly True’ gets the ‘0’ score, and the range of scores with all five scales is between 0 and 10.

**Figure 1:**
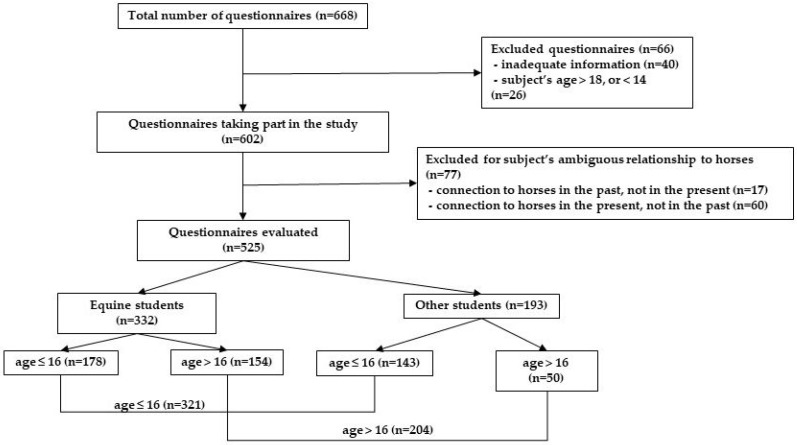
Flowchart showing the participants included in the study.

The students also filled out a general inquiry form about their age, place of living, gender, whether they owned pets, and had regular contact with horses presently or in the past. This last piece of information was necessary for the study, as we wanted to have groups that are either purely equine related or non-equine related. For this reason, students whose connection to horses was ambiguous from this point of view, i.e., other students (non-equine students) who had a relationship to horses earlier, and equine students, who had no relationship to horses in the past, were excluded. Figure 1 demonstrates the selection of the study sample.

As shown above, the total population of the present study (*n* = 525) is made up of equine students (ES) who dealt with horses in the past as well, and other (non-equine) students (OS) who had no connection to horses in the past (and none in the present, either).

#### 2.2.1. Response Variables

As mentioned earlier, the questionnaire consists of 25 statements and measures the self-reported level of five different scales: Emotional difficulties, behavioral difficulties, hyperactivity, peer-relations, and prosocial skills. The first four groups of questions concentrate on problems (the sum of these generates the total difficulty score), while the last scale, prosocial behavior, is a positive measure, giving higher points to strengths.

We analyzed the four dimensions of behavior problems and the sum of the scores of the items defining prosocial behavior as ordinal variables, and—based on the scoring instructions for SDQ [24]—categorized the scores. Since students at the vocational schools are considered to be an average, normal adolescent population, the median was chosen as the cutoff point of dichotomization. The total scores of behavior problems were considered as continuous variables, because their probability distribution was quasi normal.

#### 2.2.2. Exploratory Variables

In accordance with the goal of our research, we compare students who regularly deal with horses (equine students—ES) to other students (OS) who have no connection to horses. This way, the variable “Do you deal with horses on a regular basis?” can take two values: Yes or no.

Another exploratory variable was also introduced, namely whether students are closer to the beginning or the end of their studies. This is reflected by the dichotomous variable “Age group” with values 14–16 versus 17–18.

#### 2.2.3. Control Variable

Since there is usually a difference in behavior problems and prosocial behavior between males and females [25], gender was used as a control variable in the applied multivariable models.

### 2.3. Statistical Analysis

A frequency analysis and a chi-square test were conducted, using SPSS v. 25.0 (IBM Corp. Released 2017. IBM SPSS Statistics for Windows, Version 25.0, IBM Corp., Armonk, NY, USA) to analyze the study sample’s characteristics. A binary logistic and linear regression analysis was conducted, with reported behavior problems and prosocial behavior. We calculated odds ratios according to differences in relation to explanatory and control variables.

The K-mean cluster analysis was used to formulate two groups that are most similar to each other, considering the behavior problem scales and prosocial behavior, to verify that the basic factor differentiating between these groups is whether or not they have regular interactions with horses.

### 2.4. Ethics

The study protocols were in accordance with the latest version of the Declaration of Helsinki. The Institutional Review Board of the University of Pécs—Clinical Center, Regional and Institutional Research Ethical Committee approved the study (reference number: 7280-PTE 2018).

All participants and their parents were duly informed about the study and all provided informed consent.

## 3. Results

### 3.1. Study Sample Characteristics

Table 1 contains the number and ratio of males and females by settlement types (i.e., within settlements) among equine and other students and in the two different age groups. The Chi2 test was used to investigate whether these ratios show differences according to gender.

Table 1 shows that there are significantly more females than males in the equine groups in each of the three settlement types. Considering settlement types, gender ratios differ significantly in villages. There are relatively more females among 14–16-year-olds. Therefore, the relationship between the explanatory and predictor variables and the response variables should be controlled with the gender and age group variables. No significant relationship exists between the response variables and settlement types (city, village, farm) within the gender and age group.

Table 2 describes response variables by gender according to groups ES or OS.

Data shown in Table 2 demonstrate whether a relationship exists between behavior problems or prosocial behavior measured by the SDQ, and doing EAA, among males and females. According to the chi-square (Chi2) test, there are significantly fewer behavior difficulties and peer problems, and prosocial behavior among males is more frequent in the equine group than in the control group. In the case of female subjects, EAA seems to have a beneficial effect on the precedence of emotional difficulties and prosocial behavior.

### 3.2. Binary Logistic Regression Models

With these models, we analyzed the relationship between the exploratory variable (ES versus OS) and response variables (ED, BD, H, PR, PB) by gender and age control, using a binary logistic regression model.

The dependent variables (due to various behavior problems and prosocial behavior) of the following multiple binary logistic model (Table 3) were entered into the model in a dichotomized form, using the median. For higher score values than the median, we used code “1”, and for the lower scores, code “0”.

Table 3 shows multiple binary regression models to investigate factors that influence the occurrence of behavior problems and prosocial behavior.

The results of the table show that girls in general (i.e., independent of their age group or whether they belong to the target or the control group) have more emotional problems (AOR = 2.87; 95% CI = 1.84–4.48), and fewer peer relationship problems (AOR = 1.42; 95% CI = 0.31–0.73). Higher grade students have fewer emotional and peer problems compared to younger ones (AOR = 0.60; 95% CI = 0.41–0.91), including less hyperactivity (AOR = 0.68; 95% CI = 0.31–0.65), and their behavior is significantly more prosocial.

The most significant difference based on the SDQ questionnaire was whether the respondent belonged to the target or control group. Equine students have significantly fewer emotional and behavior problems than the average, and their prosocial behavior is significantly better. The odds of having emotional or behavioral difficulties, controlled by gender and age, are circa four times higher among non-equine students (OS) than among equine students (ES) (AOR = 4.35; 95% CI = 2.85–6.63). This suggests that EAA has a protective role, regarding behavior.

### 3.3. SDQ Total Difficulty Score

According to the SDQ evaluation description [24], the scores for the frequency of different problems can be summed up, given that the sum score can be considered as a quasi-continuous, normal distribution. This is also true in our case.

A linear regression model was used to verify the association of this variable with equine activity, controlling for gender and age (as a bivariate variable).

The results are shown in Table 4, which describes the results of the multiple linear regression model (B = unstandardized coefficients, Beta = standardized coefficients), dependent variable: Total difficulties score.

It is obvious that 17–18-year-olds have significantly (*p* < 0.001) fewer behavior problems than 14–16-year-olds, and the equine group less than the other group (*p* < 0.001). By gender (based on the partial regression coefficient), the average number of behavior problems does not differ significantly.

### 3.4. Contribution of Regular EAA to Favorable Behavior

In the following pre-post layout (our study is based on a single data collection, but we consider the design to be ‘quasi pre-post’. This is justified by the fact that during the time we conducted our research (i.e., during the past few years) there were no changes, either in the school system or in other sociological factors, that could significantly influence the choice of school or study program. It is also probable that no major difference exists in the social background of students. According to the type of their settlements (city, village, farm), where they have spent most of their lives, the sample is homogenous (*p* = 0.920)), the extent to which the statistically established favorable behavior (fewer behavior problems) of the target group can be attributed to regular activities with horses is investigated.

**Figure 2:**
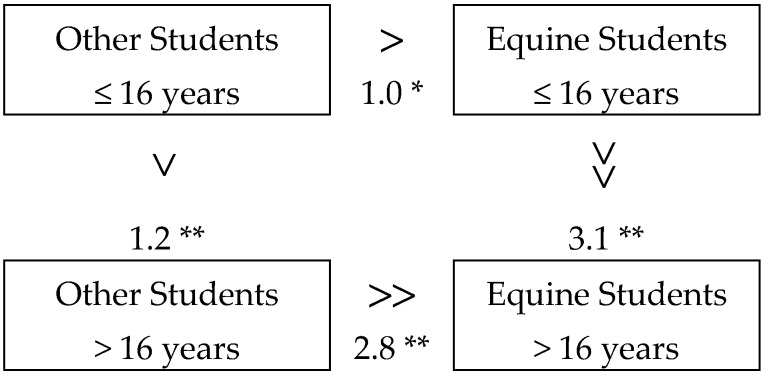
Difference of mean of the behavior problem (BP) between each group. * *p* < 0.050; ** *p* < 0.001 (asterisks (*) are according to the ANOVA Scheffe post hoc test).

In Figure 2, the relational marks show the difference of mean scores (total BP) of 14–16 and 17–18 year-olds, comparing equine and non-equine (other) student groups; the figure also compares the same average scores of equine and other students of the two different age groups.

Already upon admission to the institution (14–16 year-olds), equine students had on average fewer behavior problems than other students (difference in mean score: 1.0), and in both groups (ES and OS) of 17–18 year-olds, there were fewer behavior problems than in the group of 14–16 year-olds; however, the rate of decline is more significant among equine students (3.1 vs. 1.2).

This provides evidence that horse-related activities play a role in positive behavior changes; in other relevant respects, the vocational school students do not differ in statistical terms. It is understandable that there is a difference between ES and OS groups from the very beginning of the training, since members of the ES group have all dealt with horses before starting school. This also confirms the hypothesis that human–animal interactions contribute to behavioral development.

### 3.5. Correlation between Prosocial Behavior and Behavior Problems

In the overall sample, the total difficulty score and the prosocial scale score correlate negatively. A Spearman’s Rho = −0.30, *p* < 0.001.

Prosocial behavior (PB) was considered a dichotomous dependent variable (code: ≤3 = 0, 4+ = 1), and with a logistic regression model, we investigated to what extent it is related to the different types of behavior problems, controlled by gender, age, and group variables (ES or OS). 

Table 5 demonstrates that, except for peer relationship problems, there is a correlation between all behavior problems and prosocial behavior. The more behavior problems subjects have, the less they behave in a prosocial way. The table also demonstrates that equine students, independently of behavior problems (controlled by gender and age), are 4.6 times (AOR = 4.57, 95% CI = 2.9–7.1) more likely to behave in a prosocial way than students in the control group.

### 3.6. Clusters of Students Based on Their Answers to the SDQ

We also explored behavioral factors in other approaches, combining cluster analysis and logistic regression analysis. Table 6 shows the characteristics of the two respondent groups based on the five dimensions covered by the SDQ questionnaire (ED, BD, H, PR, PB scale scores).

As shown in Table 6, according to these variables, the two clusters are significantly (*p* < 0.001) different, with Cluster 2 clearly having a higher incidence of behavior problems and displaying less prosocial behavior than those in Cluster 1.

### 3.7. Probability of Being Included in the “Favorable” Cluster

In addition to gender, age group, and equine-related activities, the variables type of residence and pet owner were also added to the predictor variables of the logistic regression analysis, in order to find out whether these factors play a role in the behavior of our subjects.

Table 7 shows that if these new factors are also included in the investigation, gender is no longer significant, but age group (i.e., whether the study participant is at the beginning or at the end of the program) and equine-related activities are highly significant (*p* < 0.001). In the case of students belonging to the ES group, it is 2.5 times more probable (OR = 2.5; 95% CI = 1.6–3.9) that they are included in Cluster 1: That is, in the “favorable” cluster.

## 4. Discussion

Our hypothesis—that adolescents who have regular contact with, and a strong relationship to horses have fewer behavior problems and stronger prosocial skills than their peers without such engagements—is justified by the above discussed statistical results. The findings of our study have demonstrated that equine students have significantly fewer emotional and behavior problems and much stronger prosocial skills than students in the control group. It was also found that these favorable characteristics are already present at the admission of equine students to the institutions, which might suggest that adolescents with stronger social skills are attracted to horses; on the other hand, the fact that the decline of behavior problems is more remarkable in the equine group than in the control group suggests that EAA might play a role in strengthening these skills. Cluster analysis was used to show that equine-related activities represent a definitely significant factor leading to these favorable behavior traits.

It is important to mention that these beneficial effects of EAA are mostly based on the students’ understanding of and susceptibility to equine communication. The mere presence of a horse is less likely to be effective, if the equine professional present does not give meaning to the horse’s behavior. Students have to learn to treat the horses as subjects and not as objects in order to get involved and become receptive to positive influence within the interaction [9]. At the same time, this knowledge (i.e., understanding equine communication and behavior) is also essential just to be able to work safely and effectively with these animals. This means that no therapeutic goals are needed to teach students to pay attention to and respect horses—it is the basis of all equine interactions in professional environments. That is why the standard (i.e., not therapeutic or in any way specialized) school environments that we investigated could produce the above detailed results. 

As a side thought, it is probably worth mentioning that we agree with the idea of grooming as an effective way of getting attuned to the horse [26], and the fact that the to-be grooms regularly did grooming and ground work besides riding and carriage driving as part of their education could have facilitated their involvement in EAA.

It was also found that prosocial behavior became stronger with age, both in the target and control groups, although according to a previous, longitudinal study on adolescents in general, prosocial behavior decreases after mid-adolescence [27]. Other studies, related to EAA, are in line with our results and found that horse–human interactions may promote behavioral development [28,29,30], improve self-awareness and social skills [23], and cause a reduction in antisocial behavior [31]. We strongly believe that the relationship humans build with horses shows them a way to build trust, acceptance, and understanding toward humans, as well. Our results suggest that young people who learn to listen to and take care of the horse can transfer this knowledge to intraspecies communication and behavior, as well. Equine students’ prosocial behavior is four times better than that of non-equine students. This result is remarkable and supports the idea that being around horses improves students’ social competences. An earlier study found that equine therapy increases the overall level of social functioning of adolescents [32], and this study demonstrated that even without therapeutic circumstances and goals, the presence of horses has a beneficial effect.

As mentioned in the introduction, adolescence is a difficult period in life and adolescents have to cope with many difficulties during these years. They need help to understand and find their place in the world, or to just generally get around successfully. The lucky ones get enough support from their family and friends, others—a very limited number—get professional help with more serious problems. Our study showed that with a little care and attention, normal school programs can improve competencies that are useful in life. Considering the high costs of specialized programs, identifying possible beneficial means or factors that actually already exist may be of importance. If horses can be used to help adolescents and there are schools with horses and adolescents, why not exploit the possibility? With a little investment, gains might be great. We believe that besides the statistically significant scientific results indicating that horses are good for young people, this is also an important recognition of our work that might be useful for a wider, nonscientific audience, as well.

In 2015, the area of EAA was still claimed to be under-researched [33], and although the number of studies has significantly grown since then, there are always new perspectives to be discovered and new goals to be reached. More controlled circumstances and uniform methods would produce more reliable and comparable results. 

The weakness of this research consists of it not being a follow-up investigation. Data-gathering interviews occurred within a cross-sectional design, among students both at the beginning and at the end of their studies. A further weakness of the study is it failed to demonstrate in detail that during the surveyed time period, no societal-economical change took place in Hungary that would have significantly influenced the students’ recruitment or their career choice; at the same time, the study utilized this presupposition when making so-called pre-post type comparisons. 

In the study’s first half, it was established that statistically traceable differences exist between the equine and non-equine group (control group) with respect to certain types of behavior problems, as well as prosocial behavior. However, the role of animal-assisted activities in this phenomenon would only be possible in the context of a longitudinal design that allowed a reliable inquiry. Since implementing such a research would require several years, and moreover, anonymity is difficult to realize, “out of sheer necessity”, a quasi pre-post design was developed. The entire sample was arranged into two age groups, and within these, the younger age group constituted the “pre” samples, while the older age group depicted the “post” samples. 

Among the 17–18-year-olds, differences between equine and non-equine groups were enhanced, and—considering that in all other significant aspects, these groups displayed no difference—it is a viable supposition that equine training programs have a role in the positive shift of these students’ behavior.

The study’s strength is that the equine students, as well as the subjects of the control group, originate from all the educational institutions in Hungary wherein equine training programs are conducted, and even in the (entirely equine/non-equine) sample narrowed down for statistical analysis, the number of sample items remains relatively large. 

Its further strength lies in its analyses, using various approaches and multivariable models, of how equine-assisted activities affect adolescents’ prosocial behavior. Within a novel approach, it also distributes the pupils into groups based on their responses to behavior problems (with an automatic classification). One of these two significantly separate clusters gathered students with a positive behavior, while the other group included the negatives. Furthermore, utilizing a multiple logistic regression analysis, the study determines that the probability of belonging to a positive cluster is best enhanced as a result of belonging to the equine group.

## 5. Conclusions

To sum up the above, the most important results of our investigation are the following: Equine students have fewer emotional and behavior problems, and their prosocial behavior is significantly—about four times—better than that of the control group. Equine students have fewer behavior problems already upon admission to the institution (all of them had regular contact with horses before), and the rate of decline in these problems is more significant than in the other group. The total difficulty score and the prosocial scale score correlate negatively. Considering the scores referring to the different types of behavior problems and prosocial behavior all together, the difference between equine and non-equine students—independently of gender and age—is even more remarkable.

These results indicate that in this sense EAA has a protective effect on the behavior of adolescents.

Another outcome of our investigation we would like to highlight is that these results also show that equine vocational schools or programs have—to the best of our knowledge—so far unidentified potential to help adolescents with behavior problems, or possibly to prevent their development.

## Figures and Tables

**Table 1:** Characteristics by gender and settlement type.

Settlement Type	Variable	Category	All		Gender				Chi2 Sig
					Male		Female		
			*n*	%	*n*	%	*n*	%	
			525	100.0	152	29.0	373	71.0	
City	Group	Other Students	119	45.2	57	47.9	62	52.1	34.7
		Equine Students	144	54.8	21	14.6	123	85.4	<0.001
	Age (years)	14–16	163	62.0	47	28.8	116	71.2	0.1
		17–18	100	38.0	31	31.0	69	69.0	0,709
Village	Group	Other Students	41	19.5	22	53.7	19	46.3	18.1
		Equine Students	169	80.5	35	61.4	134	87.6	<0.001
	Age (years)	14–16	127	60.5	25	19.7	102	80.3	9.0
		17–18	83	39.5	32	38.5	51	61.4	0.003
Farm	Group	Other Students	33	63.5	14	42.4	19	57.6	3.9
		Equine Students	19	36.5	3	15.8	16	84.2	0.049
	Age (years)	14–16	31	59.6	11	35.5	20	64.5	0.3
		17–18	21	40.4	6	28.6	15	71.4	0.602

**Table 2:** Response variables by gender in groups equine students and other students.

Variable	Category	Male					Female				
		Other Students (*n* = 93)		Equine Students (*n* = 59)		Chi2 Sig	Other Students (*n* = 100)		Equine Students (*n* = 273)		Chi2 Sig
		*n*	Col%	*n*	Col%		*n*	Col%	*n*	Col%	
Emotional difficulties	≤3	61	65.6	46	78.0	2.7 0.103	38	38.0	146	53.5	7.0 0.008
	4+	32	34.4	13	22.0		62	62.0	127	46.5	
Behavior difficulties	≤2	28	30.1	42	71.2	24.5 <0.001	61	61.0	156	57.1	0.5 0.504
	3+	65	69.9	17	28.8		39	39.0	117	42.9	
Hyper-activity	≤3	47	50.5	36	61.0	1.6 0.206	47	47.0	149	54.6	1.7 0.194
	4+	46	49.5	23	39.0		53	53.0	124	45.4	
Peer problems	≤2	22	23.7	33	55.9	16.3 <0.001	56	56.0	152	55.7	0.003 0.956
	3+	71	76.3	26	44.1		44	44.0	121	44.3	
Prosocial behavior	≤7	74	79.6	23	39.0	25.8 <0.001	72	72.0	100	36.6	36.8 <0.001
	8+	19	20.4	36	61.0		28	28.0	173	63.4	

**Table 3:** Factors influencing the occurrence of behavior problems and prosocial behavior.

Variable	Category	Adjusted Odds Ratio (95% CI)				
		Emotional Difficulties	Behavioral Difficulties	Hyperactivity	Peer Relationship Problems	Prosocial Behavior
Gender	Female (=1)	2.87 (1.84–4.48) **	0.68 (0.45–1.03)	1.06 (0.70–1.61)	0.48 (0.31–0.73) **	1.42 (0.91–2.22)
	Male (=0)	Reference	Reference	Reference	Reference	Reference
Age (year)	17–18 (=1)	0.60 (0.41–0.88) **	0.79 (0.55–1.15)	0.45 (0.31–0.65) **	0.68 (0.47–0.99) *	1.79 (1.21–2.65) **
	14–16 (=0)	Reference	Reference	Reference	Reference	Reference
Group	ES (=1)	0.60 (0.40–0.91) *	0.68 (0.46–0.99) *	0.87 (0.58–1.29)	0.72 (0.48–1.06)	4.35 (2.85–6.63) **
	OS (=0)	Reference	Reference	Reference	Reference	Reference

ES: Equine students; OS: Other students. * *p* < 0.05; ** *p* < 0.01.

**Table 4:** Results of the multiple linear regression model.

Variable	B	SE	Beta	t	Sig
**(Constant)**	14.162	0.471		30.057	<0.001
**Gender** **(male = 0, female = 1)**	−0.105	0.511	−0.009	−0.206	0.837
**Age group (years)** **(14–16 = 0, 17–18 = 1)**	−2.293	0.46	−0.216	−4.99	<0.001
**Group** **(ES = 1, OS = 0)**	−1.785	0.489	−0.166	−3.65	<0.001

Dependent variable: Total difficulty score; ES: Equine students; OS: Other students.

**Table 5:** Relationship between prosocial behavior (dependent variable) and behavior problems, with a logistic regression model.

Variable	B	SE	Sig	Exp(B)	95% CI for EXP(B)	
					Lower	Upper
Emotional difficulties (≤3 = 1, 4+ = 2)	−0.587	0.215	0.006	0.555	0.390	0.827
Behavioral difficulties (≤2 = 1, 3+ = 2)	−0.586	0.207	0.005	0.557	0.371	0.834
Hyperactivity (≤3 = 1, 4+ = 2)	−0.550	0.209	0.008	0.577	0.383	0.869
Peer-relations (≤2 = 1, 3+ = 2)	−0.317	0.205	0.123	0.729	0.487	1.090
Gender (male = 0, female = 1)	0.158	0.243	0.516	1.171	0.728	1.884
Age group (years) (14–16 = 0, 17–18 = 1)	0.508	0.208	0.015	1.661	1.104	2.500
Group (Equine students = 1, Other students = 0)	1.518	0.223	<0.001	4.565	2.946	7.072
Constant	−0.078	0.536	0.884	0.925		

**Table 6:** Descriptions of response variables by cluster.

Response Variables	Cluster 1 (*n* = 277)		Cluster 2 (*n* = 248)	
	Mean	SD	Mean	SD
Emotional difficulties	2.27	1.74	4.15	2.30
Behavioral difficulties	1.66	0.99	3.42	1.69
Hyperactivity	2.27	1.45	4.65	1.87
Peer relationship problems	1.96	1.36	3.35	1.97
Prosocial behavior	8.40	1.41	6.22	1.88

**Table 7:** Factors influencing the probability of belonging to Cluster 1, with a logistic regression model.

Variable	B	SE	Sig	Exp(B)	95% CI for EXP(B)	
					Lower	Upper
Gender (male = 0, female = 1)	0.110	0.222	0.619	1.117	0.723	1.725
Age group (years) (14–16 = 0, 17–18 = 1)	0.809	0.195	<0.001	2.246	1.534	3.288
Type of residence			0.595			
Village vs. City	−0.333	0.327	0.308	0.717	0.378	1.360
Farm vs. City	−0.287	0.342	0.401	0.750	0.384	1.467
Pet owner (yes = 1, no = 0)	0.376	0.313	0.230	1.456	0.789	2.690
Group (ES = 1, OS = 0)	0.923	0.223	<0.001	2.516	1.624	3.897
Constant	−1.156	0.434	0.008	0.315	0.723	1.725

Dependent variable: Cluster 1 (=1) vs. Cluster 2 (=0); ES: Equine students; OS: Other students.
